# Supplementary material for: Recognising and evaluating the effectiveness of extortion in the Iterated Prisoner’s Dilemma
Source: PLoS One. 2024 Jul 26;19(7):e0304641. doi: 10.1371/journal.pone.0304641 (PMC11280246; doi:10.1371/journal.pone.0304641)
Supplement: S1 Appendix — (PDF) [file pone.0304641.s001.pdf]

Proof of algebraic condition for extortionate strategies

The defining equations for an extortionate strategy are:

$$\tilde{p}_1 = \alpha(R - P) + \beta(R - P) \tag{1}$$
$$\tilde{p}_2 = \alpha(S - P) + \beta(T - P) \tag{2}$$
$$\tilde{p}_3 = \alpha(T - P) + \beta(S - P) \tag{3}$$
$$\tilde{p}_4 = 0 \tag{4}$$

Using equation (2), α is isolated

$$\alpha = \frac{-\beta(P - T) - \tilde{p}_2}{P - S} \tag{5}$$

Substituting this value in to equation (3), β is isolated:

$$\beta = -\frac{P\tilde{p}_1 - P\tilde{p}_2 + S\tilde{p}_2 - T\tilde{p}_1}{(S - T)(2P - S - T)} \tag{6}$$

Substituting this back in to (5) gives:

$$\alpha = \frac{-\tilde{p}_2 + (P - T)(P\tilde{p}_1 - P\tilde{p}_2 + S\tilde{p}_2 - T\tilde{p}_1)}{(S - T)(2P - S - T)(P - S)} \tag{7}$$

Substituting equations (6-7) in to equation (1) gives the required expression for p_1.
Taking the ratio of equations (6-7) gives the required expression for χ.
Finally, the condition $\chi > 1$ corresponds to:

$$\tilde{p}_2(P - T) + \tilde{p}_3(S - P) > \tilde{p}_2(P - S) + \tilde{p}_3(T - P) \tag{8}$$

which can be simplified to:

$$\tilde{p}_2 < -\tilde{p}_3 \tag{9}$$

recalling that $\tilde{p}_2 = p_2 - 1$ and $\tilde{p}_3 = p_3$ gives the required result.
