# Supplementary figures and images for: Recognising and evaluating the effectiveness of extortion in the Iterated Prisoner’s Dilemma
Source: PLoS One. 2024 Jul 26;19(7):e0304641. doi: 10.1371/journal.pone.0304641 (PMC11280246; doi:10.1371/journal.pone.0304641)

Skew of SSE sorted by score

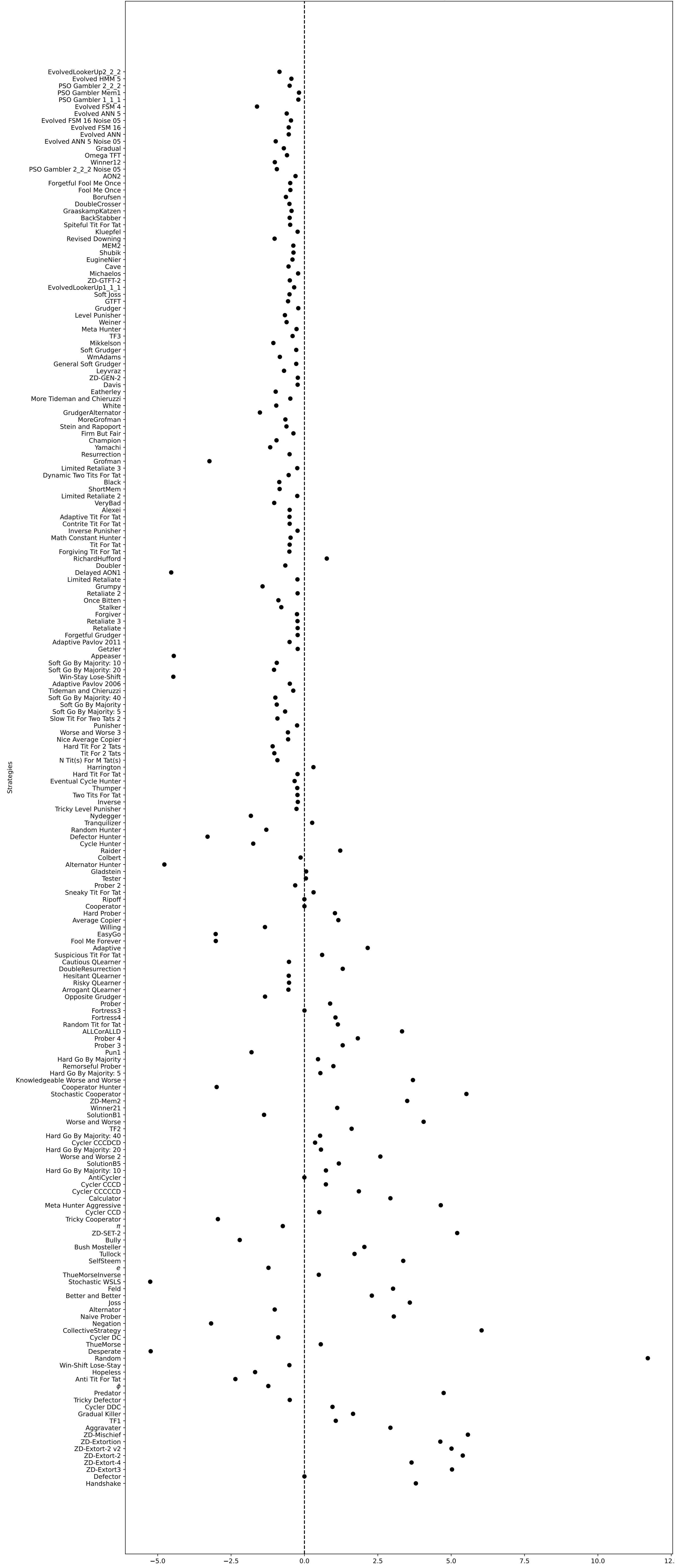

Skew of SSE sorted by number of wins

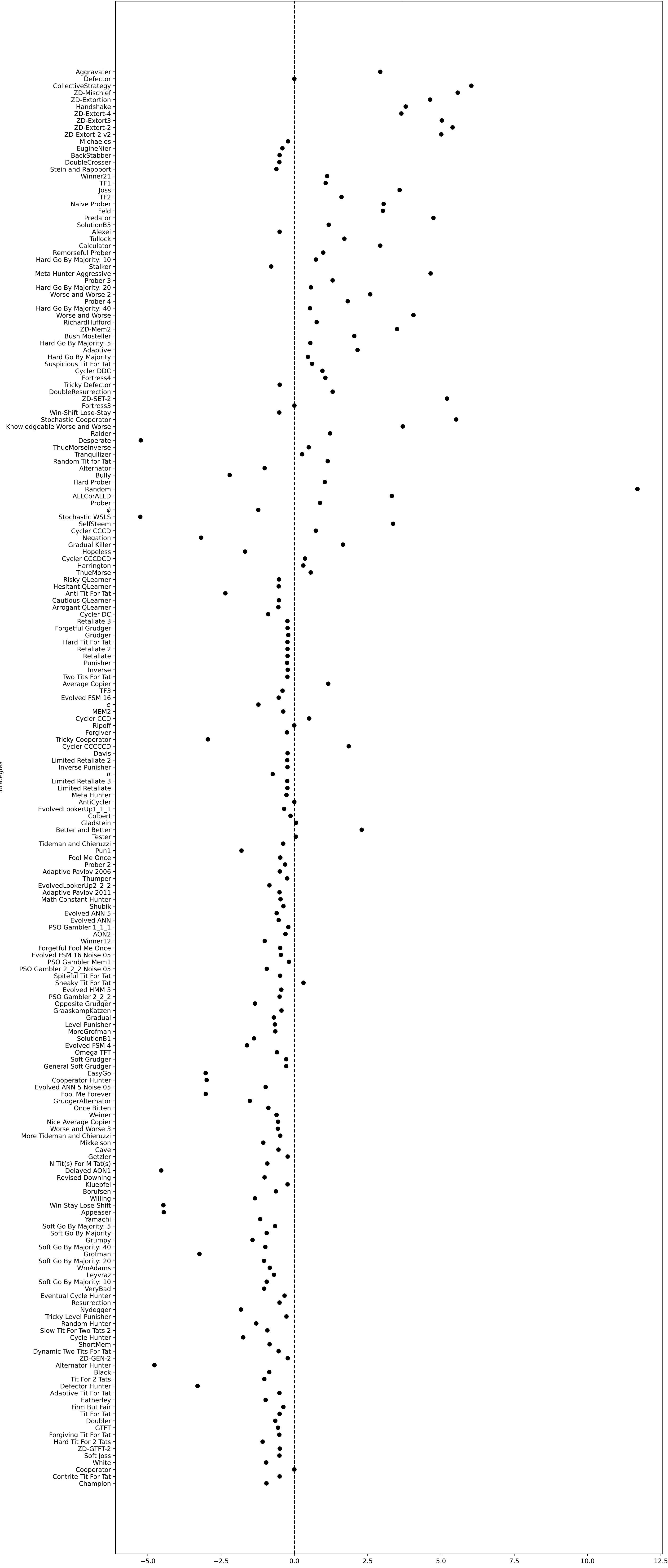

Supplement: S2 Appendix — The skew for all the strategies in the larger tournament. (PDF) [file pone.0304641.s002.pdf]
