# Supplementary material for: Recognising and evaluating the effectiveness of extortion in the Iterated Prisoner’s Dilemma
Source: PLoS One. 2024 Jul 26;19(7):e0304641. doi: 10.1371/journal.pone.0304641 (PMC11280246; doi:10.1371/journal.pone.0304641)
Supplement: S3 Appendix — (PDF) [file pone.0304641.s003.pdf]

List of all strategies used from [21]

1. Adaptive - Deterministic - Memory length: ∞ - [25]
2. Adaptive Tit For Tat: 0.5 - Deterministic - Memory length: ∞ - [39]
3. Aggravater - Deterministic - Memory length: ∞ - [21]
4. Alexei: (D,) - Deterministic - Memory length: ∞ - [43]
5. ALLCorALLD - Stochastic - Memory length: 1 - [2]
6. Alternator - Deterministic - Memory length: 1 - [11, 31]
7. Alternator Hunter - Deterministic - Memory length: ∞ - [21]
8. AntiCycler - Deterministic - Memory length: ∞ - [21]
9. Anti Tit For Tat - Deterministic - Memory length: 1 - [18]
10. AON2 - Deterministic - Memory length: 2 - [19]
11. Adaptive Pavlov 2006 - Deterministic - Memory length: ∞ - [24]
12. Adaptive Pavlov 2011 - Deterministic - Memory length: ∞ - [25]
13. Appeaser - Deterministic - Memory length: ∞ - [21]
14. Arrogant QLearner - Stochastic - Memory length: ∞ - [21]
15. Average Copier - Stochastic - Memory length: ∞ - [21]
16. BackStabber: (D, D) - Deterministic - Memory length: ∞ - [21]
17. Better and Better - Stochastic - Memory length: ∞ - [29]
18. Black - Stochastic - Memory length: 5 - [10]
19. Borufsen - Deterministic - Memory length: ∞ - [10]
20. Bully - Deterministic - Memory length: 1 - [32]
21. Bush Mosteller: 0.5, 0.5, 3.0, 0.5 - Stochastic - Memory length: ∞ - [20]
22. Calculator - Stochastic - Memory length: ∞ - [29]
23. Cautious QLearner - Stochastic - Memory length: ∞ - [21]
24. Cave - Stochastic - Memory length: ∞ - [10]
25. Champion - Stochastic - Memory length: ∞ - [10]
26. Colbert - Deterministic - Memory length: 4 - [10]
27. CollectiveStrategy - Deterministic - Memory length: ∞ - [26]
28. Contrite Tit For Tat - Deterministic - Memory length: 3 - [42]
29. Cooperator - Deterministic - Memory length: 0 - [11, 31, 34]
30. Cooperator Hunter - Deterministic - Memory length: ∞ - [21]
31. Cycle Hunter - Deterministic - Memory length: ∞ - [21]
32. Cycler CCCCCD - Deterministic - Memory length: 5 - [21]
33. Cycler CCCD - Deterministic - Memory length: 3 - [21]
34. Cycler CCD - Deterministic - Memory length: 2 - [31]
35. Cycler DC - Deterministic - Memory length: 1 - [21]
36. Cycler DDC - Deterministic - Memory length: 2 - [31]
37. Cycler CCCDCD - Deterministic - Memory length: 5 - [21]
38. Davis: 10 - Deterministic - Memory length: ∞ - [9]
39. Defector - Deterministic - Memory length: 0 - [11, 31, 34]
40. Defector Hunter - Deterministic - Memory length: ∞ - [21]
41. Desperate - Stochastic - Memory length: 1 - [41]
42. Delayed AON1 - Deterministic - Memory length: 2 - [19]
43. DoubleCrosser: (D, D) - Deterministic - Memory length: ∞ - [21]
44. Doubler - Deterministic - Memory length: ∞ - [29]
45. DoubleResurrection - Deterministic - Memory length: 5 - [15]
46. EasyGo - Deterministic - Memory length: ∞ - [29, 25]
47. Eatherley - Stochastic - Memory length: ∞ - [10]
48. EugineNier: (D,) - Deterministic - Memory length: ∞ - [43]
49. Eventual Cycle Hunter - Deterministic - Memory length: ∞ - [21]
50. Evolved ANN - Deterministic - Memory length: ∞ - [21]
51. Evolved ANN 5 - Deterministic - Memory length: ∞ - [21]
52. Evolved ANN 5 Noise 05 - Deterministic - Memory length: ∞ - [21]
53. Evolved FSM 4 - Deterministic - Memory length: ∞ - [21]
54. Evolved FSM 16 - Deterministic - Memory length: ∞ - [21]
55. Evolved FSM 16 Noise 05 - Deterministic - Memory length: ∞ - [21]
56. EvolvedLookerUp1_1_1 - Deterministic - Memory length: ∞ - [21]
57. EvolvedLookerUp2_2_2 - Deterministic - Memory length: ∞ - [21]
58. Evolved HMM 5 - Stochastic - Memory length: 5 - [21]
59. Feld: 1.0, 0.5, 200 - Stochastic - Memory length: 200 - [9]
60. Firm But Fair - Stochastic - Memory length: 1 - [16]
61. Fool Me Forever - Deterministic - Memory length: ∞ - [21]
62. Fool Me Once - Deterministic - Memory length: ∞ - [21]
63. Forgetful Fool Me Once: 0.05 - Stochastic - Memory length: ∞ - [21]
64. Forgetful Grudger - Deterministic - Memory length: 10 - [21]
65. Forgiver - Deterministic - Memory length: ∞ - [21]
66. Forgiving Tit For Tat - Deterministic - Memory length: ∞ - [21]
67. Fortress3 - Deterministic - Memory length: 2 - [7]
68. Fortress4 - Deterministic - Memory length: 3 - [7]
69. GTFT: 0.33 - Stochastic - Memory length: 1 - [33, 17]
70. General Soft Grudger: n=1,d=4,c=2 - Deterministic - Memory length: ∞ - [21]
71. Getzler - Stochastic - Memory length: ∞ - [10]
72. Gladstein - Deterministic - Memory length: ∞ - [10]
73. Soft Go By Majority - Deterministic - Memory length: ∞ - [11, 31, 10]
74. Soft Go By Majority: 10 - Deterministic - Memory length: 10 - [21]
75. Soft Go By Majority: 20 - Deterministic - Memory length: 20 - [21]
76. Soft Go By Majority: 40 - Deterministic - Memory length: 40 - [21]

77. Soft Go By Majority: 5 - Deterministic - Memory length: 5 - [21]
78. ϕ - Deterministic - Memory length: ∞ - [21]
79. GraaskampKatzen - Deterministic - Memory length: ∞ - [10]
80. Gradual - Deterministic - Memory length: ∞ - [13]
81. Gradual Killer: (D, D, D, D, D, C, C) - Deterministic - Memory length: ∞ - [29]
82. Grofman - Stochastic - Memory length: ∞ - [9]
83. Grudger - Deterministic - Memory length: 1 - [25, 9, 41, 12, 13]
84. GrudgerAlternator - Deterministic - Memory length: ∞ - [29]
85. Grumpy: Nice, 10, -10 - Deterministic - Memory length: ∞ - [21]
86. Handshake - Deterministic - Memory length: ∞ - [35]
87. Hard Go By Majority - Deterministic - Memory length: ∞ - [31]
88. Hard Go By Majority: 10 - Deterministic - Memory length: 10 - [21]
89. Hard Go By Majority: 20 - Deterministic - Memory length: 20 - [21]
90. Hard Go By Majority: 40 - Deterministic - Memory length: 40 - [21]
91. Hard Go By Majority: 5 - Deterministic - Memory length: 5 - [21]
92. Hard Prober - Deterministic - Memory length: ∞ - [29]
93. Hard Tit For 2 Tats - Deterministic - Memory length: 3 - [38]
94. Hard Tit For Tat - Deterministic - Memory length: 3 - [40]
95. Harrington - Stochastic - Memory length: ∞ - [10]
96. Hesitant QLearner - Stochastic - Memory length: ∞ - [21]
97. Hopeless - Stochastic - Memory length: 1 - [41]
98. Inverse - Stochastic - Memory length: ∞ - [21]
99. Inverse Punisher - Deterministic - Memory length: ∞ - [21]
100. Joss: 0.9 - Stochastic - Memory length: 1 - [38, 9]
101. Kluepfel - Stochastic - Memory length: ∞ - [10]
102. Knowledgeable Worse and Worse - Stochastic - Memory length: ∞ - [21]
103. Level Punisher - Deterministic - Memory length: ∞ - [15]
104. Leyvraz - Stochastic - Memory length: 3 - [10]
105. Limited Retaliate: 0.1, 20 - Deterministic - Memory length: ∞ - [21]
106. Limited Retaliate 2: 0.08, 15 - Deterministic - Memory length: ∞ - [21]
107. Limited Retaliate 3: 0.05, 20 - Deterministic - Memory length: ∞ - [21]
108. Math Constant Hunter - Deterministic - Memory length: ∞ - [21]
109. Naive Prober: 0.1 - Stochastic - Memory length: 1 - [25]
110. MEM2 - Deterministic - Memory length: ∞ - [27]
111. Michaelos: (D,) - Stochastic - Memory length: ∞ - [43]
112. Mikkelson - Deterministic - Memory length: ∞ - [10]
113. MoreGrofman - Deterministic - Memory length: 8 - [10]
114. More Tideman and Chieruzzi - Deterministic - Memory length: ∞ - [10]
115. Negation - Stochastic - Memory length: 1 - [40]
116. Nice Average Copier - Stochastic - Memory length: ∞ - [21]
117. N Tit(s) For M Tat(s): 3, 2 - Deterministic - Memory length: 3 - [21]
118. Nydegger - Deterministic - Memory length: 3 - [9]
119. Omega TFT: 3, 8 - Deterministic - Memory length: ∞ - [37]
120. Once Bitten - Deterministic - Memory length: 12 - [21]
121. Opposite Grudger - Deterministic - Memory length: ∞ - [21]
122. π - Deterministic - Memory length: ∞ - [21]
123. Predator - Deterministic - Memory length: ∞ - [7]
124. Prober - Deterministic - Memory length: ∞ - [25]
125. Prober 2 - Deterministic - Memory length: ∞ - [29]
126. Prober 3 - Deterministic - Memory length: ∞ - [29]
127. Prober 4 - Deterministic - Memory length: ∞ - [29]
128. Pun1 - Deterministic - Memory length: ∞ - [6]
129. PSO Gambler 1_1_1 - Stochastic - Memory length: ∞ - [21]
130. PSO Gambler 2_2_2 - Stochastic - Memory length: ∞ - [21]
131. PSO Gambler 2_2_2 Noise 05 - Stochastic - Memory length: ∞ - [21]
132. PSO Gambler Mem1 - Stochastic - Memory length: 1 - [21]
133. Punisher - Deterministic - Memory length: ∞ - [21]
134. Raider - Deterministic - Memory length: ∞ - [8]
135. Random: 0.5 - Stochastic - Memory length: 0 - [39, 9]
136. Random Hunter - Deterministic - Memory length: ∞ - [21]
137. Random Tit for Tat: 0.5 - Stochastic - Memory length: 1 - [21]
138. Remorseful Prober: 0.1 - Stochastic - Memory length: 2 - [25]
139. Resurrection - Deterministic - Memory length: 5 - [15]
140. Retaliate: 0.1 - Deterministic - Memory length: ∞ - [21]
141. Retaliate 2: 0.08 - Deterministic - Memory length: ∞ - [21]
142. Retaliate 3: 0.05 - Deterministic - Memory length: ∞ - [21]
143. Revised Downing: True - Deterministic - Memory length: ∞ - [9]
144. RichardHufford - Deterministic - Memory length: ∞ - [10]
145. Ripoff - Deterministic - Memory length: 3 - [5]
146. Risky QLearner - Stochastic - Memory length: ∞ - [21]
147. SelfSteem - Stochastic - Memory length: ∞ - [14]
148. ShortMem - Deterministic - Memory length: 10 - [14]
149. Shubik - Deterministic - Memory length: ∞ - [9]
150. Slow Tit For Two Tats 2 - Deterministic - Memory length: 2 - [29]
151. Sneaky Tit For Tat - Deterministic - Memory length: ∞ - [21]
152. Soft Grudger - Deterministic - Memory length: 6 - [25]
153. Soft Joss: 0.9 - Stochastic - Memory length: 1 - [29]
154. SolutionB1 - Deterministic - Memory length: 2 - [4]
155. SolutionB5 - Deterministic - Memory length: ∞ - [4]
156. Spiteful Tit For Tat - Deterministic - Memory length: ∞ - [29]
157. Stalker: (D,) - Stochastic - Memory length: ∞ - [14]
158. Stein and Rapoport: 0.05: (D, D) - Deterministic - Memory length: ∞ - [9]
159. Stochastic Cooperator - Stochastic - Memory length: 1 - [1]
160. Stochastic WSLS: 0.05 - Stochastic - Memory length: 1 - [3]
161. Suspicious Tit For Tat - Deterministic - Memory length: 1 - [13, 18]
162. Tester - Deterministic - Memory length: ∞ - [10]

163. TF1 - Deterministic - Memory length: ∞ - [21]
164. TF2 - Deterministic - Memory length: ∞ - [21]
165. TF3 - Deterministic - Memory length: ∞ - [21]
166. ThueMorse - Deterministic - Memory length: ∞ - [21]
167. ThueMorseInverse - Deterministic - Memory length: ∞ - [21]
168. Thumper - Deterministic - Memory length: ∞ - [5]
169. Tideman and Chieruzzi - Deterministic - Memory length: ∞ - [9]
170. Tit For Tat - Deterministic - Memory length: 1 - [9]
171. Tit For 2 Tats - Deterministic - Memory length: 2 - [11]
172. Tranquilizer - Stochastic - Memory length: ∞ - [9]
173. Tricky Cooperator - Deterministic - Memory length: 10 - [21]
174. Tricky Defector - Deterministic - Memory length: ∞ - [21]
175. Tricky Level Punisher - Deterministic - Memory length: ∞ - [15]
176. Tullock: 11 - Stochastic - Memory length: 11 - [9]
177. Two Tits For Tat - Deterministic - Memory length: 2 - [11]
178. VeryBad - Deterministic - Memory length: ∞ - [14]
179. Weiner - Deterministic - Memory length: ∞ - [10]
180. White - Deterministic - Memory length: ∞ - [10]
181. Willing - Stochastic - Memory length: 1 - [41]
182. Winner12 - Deterministic - Memory length: 2 - [30]
183. Winner21 - Deterministic - Memory length: 2 - [30]
184. Win-Shift Lose-Stay: D - Deterministic - Memory length: 1 - [25]
185. Win-Stay Lose-Shift: C - Deterministic - Memory length: 1 - [33, 38, 22]
186. WmAdams - Stochastic - Memory length: ∞ - [10]
187. Worse and Worse - Stochastic - Memory length: ∞ - [29]
188. Worse and Worse 2 - Stochastic - Memory length: ∞ - [29]
189. Worse and Worse 3 - Stochastic - Memory length: ∞ - [29]
190. Yamachi - Deterministic - Memory length: ∞ - [10]
191. ZD-Extortion: 0.2, 0.1, 1 - Stochastic - Memory length: 1 - [36]
192. ZD-Extort-2: 0.1111111111111111, 0.5 - Stochastic - Memory length: 1 - [38]
193. ZD-Extort3: 0.11538461538461539, 0.3333333333333333, 1 - Stochastic - Memory length: 1 - [34]
194. ZD-Extort-2 v2: 0.125, 0.5, 1 - Stochastic - Memory length: 1 - [23]
195. ZD-Extort-4: 0.23529411764705882, 0.25, 1 - Stochastic - Memory length: 1 - [21]
196. ZD-GTFT-2: 0.25, 0.5 - Stochastic - Memory length: 1 - [38]
197. ZD-GEN-2: 0.125, 0.5, 3 - Stochastic - Memory length: 1 - [23]
198. ZD-Mem2 - Stochastic - Memory length: 2 - [28]
199. ZD-Mischief: 0.1, 0.0, 1 - Stochastic - Memory length: 1 - [36]
200. ZD-SET-2: 0.25, 0.0, 2 - Stochastic - Memory length: 1 - [23]
201. e - Deterministic - Memory length: ∞ - [21]
202. Dynamic Two Tits For Tat - Stochastic - Memory length: ∞ - [21]
203. Meta Hunter: 6 players - Deterministic - Memory length: ∞ - [21]
204. Meta Hunter Aggressive: 7 players - Deterministic - Memory length: ∞ - [21]

References

[1] Christoph Adami and Arend Hintze. "Evolutionary instability of zero-determinant strategies demonstrates that winning is not everything." In: *Nature communications* 4.1 (2013), p. 2193. ISSN: 2041-1723. DOI: 10.1038/ncomms3193. arXiv: arXiv:1208.2666v4. URL: http://www.pubmedcentral.nih.gov/articlerender.fcgi?artid=3741637%7B%5C&%7Dtool=pmcentrez%7B%5C&%7Drendertype=abstract.

[2] Ethan Akin. "What you gotta know to play good in the iterated prisoners dilemma". In: *Games* 6.3 (2015), pp. 175–190.

[3] Marco A Amaral et al. "Stochastic win-stay-lose-shift strategy with dynamic aspirations in evolutionary social dilemmas". In: *Physical Review E* 94.3 (2016), p. 032317.

[4] Daniel Ashlock, Joseph Alexander Brown, and Philip Hingston. "Multiple Opponent Optimization of Prisoners Dilemma Playing Agents". In: *IEEE Transactions on Computational Intelligence and AI in Games* 7.1 (2015), pp. 53–65.

[5] Daniel Ashlock and Eun-Youn Kim. "Fingerprinting: Visualization and automatic analysis of prisoner's dilemma strategies". In: *IEEE Transactions on Evolutionary Computation* 12.5 (2008), pp. 647–659.

[6] Daniel Ashlock, Eun-Youn Kim, and Nicole Leahy. "Understanding representational sensitivity in the iterated prisoner's dilemma with fingerprints". In: *IEEE Transactions on Systems, Man, and Cybernetics, Part C (Applications and Reviews)* 36.4 (2006), pp. 464–475.

[7] Wendy Ashlock and Daniel Ashlock. "Changes in prisoners dilemma strategies over evolutionary time with different population sizes". In: *Evolutionary Computation, 2006. CEC 2006. IEEE Congress on.* IEEE. 2006, pp. 297–304.

[8] Wendy Ashlock, Jeffrey Tsang, and Daniel Ashlock. "The evolution of exploitation". In: *Foundations of Computational Intelligence (FOCI), 2014 IEEE Symposium on.* IEEE. 2014, pp. 135–142.

[9] Robert Axelrod. "Effective choice in the prisoner's dilemma". In: *Journal of conflict resolution* 24.1 (1980), pp. 3–25.

[10] Robert Axelrod. "More Effective Choice in the Prisoner's Dilemma". In: *Journal of Conflict Resolution* 24.3 (1980), pp. 379–403. ISSN: 0022-0027. DOI: 10.1177/002200278002400301.

[11] Robert Axelrod. *The Evolution of Cooperation.* Basic Books, 1985. ISBN: 0-465-02121-2. URL: https://www.amazon.com/Evolution-Cooperation-Robert-Axelrod/dp/0465021212?SubscriptionId=AKIAIOBINVZYXZQZ2U3A&tag=chimbori05-20&linkCode=xm2&camp=2025&creative=165953&creativeASIN=0465021212.

[12] Jeffrey S Banks and Rangarajan K Sundaram. "Repeated games, finite automata, and complexity". In: *Games and Economic Behavior* 2.2 (1990), pp. 97–117.

[13] Bruno Beaufils, Jean-Paul Delahaye, and Philippe Mathieu. "Our meeting with gradual, a good strategy for the iterated prisoners dilemma". In: *Proceedings of the Fifth International Workshop on the Synthesis and Simulation of Living Systems.* 1997, pp. 202–209.

[14] Andre LC Carvalho et al. "Iterated Prisoners Dilemma-An extended analysis". In: (2013), pp. 1–6. DOI: 10.21528/CBIC2013-202.

[15] Arnold Eckhart. *CoopSim v0.9.9 beta 6.* https://github.com/jecki/CoopSim/. 2015.

[16] Marcus R Frean. "The prisoner's dilemma without synchrony". In: *Proceedings of the Royal Society of London B: Biological Sciences* 257.1348 (1994), pp. 75–79.

[17] Marco Gaudesi et al. "Exploiting evolutionary modeling to prevail in iterated prisoners dilemma tournaments". In: *IEEE Transactions on Computational Intelligence and AI in Games* 8.3 (2016), pp. 288–300.

[18] C. Hilbe, M. A. Nowak, and K. Sigmund. “Evolution of extortion in Iterated Prisoner’s Dilemma games”. In: *Proceedings of the National Academy of Sciences* 110.17 (Apr. 2013), pp. 6913–6918. DOI: 10.1073/pnas.1214834110.

[19] Christian Hilbe et al. “Memory-n strategies of direct reciprocity”. In: *Proceedings of the National Academy of Sciences* 114.18 (2017), pp. 4715–4720.

[20] Luis R Izquierdo and Segismundo S Izquierdo. “Dynamics of the Bush-Mosteller learning algorithm in 2x2 games”. In: *Reinforcement Learning*. InTech, 2008.

[21] Vince Knight et al. *Axelrod-Python/Axelrod: V4.2.0*. 2018. DOI: 10.5281/zenodo.1252994.

[22] David Kraines and Vivian Kraines. “Pavlov and the prisoner’s dilemma”. In: *Theory and decision* 26.1 (1989), pp. 47–79. ISSN: 00405833. DOI: 10.1007/BF00134056.

[23] Steven Kuhn. “Prisoner’s Dilemma”. In: *The Stanford Encyclopedia of Philosophy*. Ed. by Edward N. Zalta. Spring 2017. Metaphysics Research Lab, Stanford University, 2017.

[24] Jiawei Li et al. “How to design a strategy to win an IPD tournament”. In: *The iterated prisoners dilemma* 20 (2007), pp. 89–104.

[25] Jiawei Li, Philip Hingston, and Graham Kendall. “Engineering design of strategies for winning iterated prisoner’s dilemma competitions”. In: *IEEE Transactions on Computational Intelligence and AI in Games* 3.4 (2011), pp. 348–360.

[26] Jiawei Li and Graham Kendall. “A strategy with novel evolutionary features for the iterated prisoner’s dilemma.” In: *Evolutionary Computation* 17.2 (2009), pp. 257–274. ISSN: 1063-6560. DOI: 10.1162/evco.2009.17.2.257. URL: http://www.ncbi.nlm.nih.gov/pubmed/19413490.

[27] Jiawei Li and Graham Kendall. “The effect of memory size on the evolutionary stability of strategies in iterated prisoner’s dilemma”. In: *IEEE Transactions on Evolutionary Computation* 18.6 (2014), pp. 819–826. DOI: 10.1109/TEVC.2013.2286492.

[28] Siwei Li. *Strategies in the Stochastic Iterated Prisoner’s Dilemma*. http://math.uchicago.edu/ may/REU2014/REUPapers/Li,Siwei.pdf. 2014.

[29] LIFL. *PRISON*. http://www.lifl.fr/IPD/ipd.frame.html. 2008.

[30] Philippe Mathieu and Jean-Paul Delahaye. “New Winning Strategies for the Iterated Prisoner’s Dilemma (Extended Abstract)”. In: *14th International Conference on Autonomous Agents and Multiagent Systems (AAMAS 2015)* (2015), pp. 1665–1666. ISSN: 15582914.

[31] Shashi Mittal and Kalyanmoy Deb. “Optimal strategies of the iterated prisoner’s dilemma problem for multiple conflicting objectives”. In: *IEEE Transactions on Evolutionary Computation* 13.3 (2009), pp. 554–565.

[32] John H Nachbar. “Evolution in the finitely repeated prisoner’s dilemma”. In: *Journal of Economic Behavior & Organization* 19.3 (1992), pp. 307–326.

[33] Martin A Nowak and Karl Sigmund. “A strategy of win-stay, lose-shift that outperforms tit-for-tat in the Prisoner’s Dilemma game.” In: *Nature* 364.6432 (1993), pp. 56–58. ISSN: 0028-0836. DOI: 10.1038/364056a0.

[34] W. H. Press and F. J. Dyson. “Iterated Prisoner’s Dilemma contains strategies that dominate any evolutionary opponent”. In: *Proceedings of the National Academy of Sciences* 109.26 (May 2012), pp. 10409–10413. DOI: 10.1073/pnas.1206569109.

[35] Arthur J Robson. “Efficiency in evolutionary games: Darwin, Nash and the secret handshake”. In: *Journal of theoretical Biology* 144.3 (1990), pp. 379–396.

[36] Lars Roemheld. “Evolutionary Extortion and Mischief: Zero Determinant strategies in iterated 2x2 games”. In: *arXiv preprint arXiv:1308.2576* (2013).

[37] Wolfgang Slany, Wolfgang Kienreich, et al. “On some winning strategies for the Iterated Prisoners Dilemma, or, Mr. Nice Guy and the Cosa Nostra”. In: *The iterated prisoners dilemma* 20 (2007), p. 171.

[38] A. J. Stewart and J. B. Plotkin. “Extortion and cooperation in the Prisoner’s Dilemma”. In: *Proceedings of the National Academy of Sciences* 109.26 (June 2012), pp. 10134–10135. DOI: 10.1073/pnas.1208087109.

[39] Elpida Tzafestas. “Toward adaptive cooperative behavior”. In: *Proceedings of the Simulation of Adaptive Behavior Conference*. Citeseer. 2000, pp. 334–340.

[40] Unkwown. *www.prisoners-dilemma.com*. http://www.prisoners-dilemma.com/. 2017.

[41] Pieter Van den Berg and Franz J Weissing. “The importance of mechanisms for the evolution of cooperation”. In: *Proc. R. Soc. B*. Vol. 282. 1813. The Royal Society. 2015, p. 20151382.

[42] Jianzhong Wu and Robert Axelrod. “How to cope with noise in the iterated prisoner’s dilemma”. In: *Journal of Conflict resolution* 39.1 (1995), pp. 183–189.

[43] *Zoo of strategies*. 2011. URL: http://lesswrong.com/lw/7f2/prisoners_dilemma_tournament_results/.
