# Supplementary material for: Recognising and evaluating the effectiveness of extortion in the Iterated Prisoner’s Dilemma
Source: PLoS One. 2024 Jul 26;19(7):e0304641. doi: 10.1371/journal.pone.0304641 (PMC11280246; doi:10.1371/journal.pone.0304641)
Supplement: Attachment — Submitted filename: main.docx [file pone.0304641.s004.docx]

# Response to reviewers

We would like to thank both reviewers for their comments on the manscript.

We attach a point by point response to each of their points, and in the case of reviewer 1 a point by point response to each of the points raised by the editor for a previous review.

We also include a marked up document showing the changes since our original submission to PLOS One. Although we not that this was done prior to changing the style of the manuscript to match the style of PLOS One: this is to simplify how the marked up document looks. Note that this marked up document does not include changes to any of the mathematical equations.

## Reviewer 1

I reviewed this manuscript in another journal before and finally gave “Accept” in their revision 1. However, the handling editor gave “Reject” because he/she thought that the presentation of the paper is problematic.

I personally think this manuscript is worth publishing in PLOS ONE. However, at the same time, I think the points suggested by the handling editor at that time were also very reasonable. I just want to know if those points by the handling editor are clearly addressed in this submitted manuscript in PLOS ONE. If yes, please provide a detailed list of your revisions so I can check it.

We agree that the comments by the handling editor at the time were reasonable. We took on their comments and made all the required changes.

The comments from the area editor were:

- It is not clear from Figure 1 whether sorting is made in an ascending or descending order from left to right. Readers have to guess it.

This has been clarified in the caption.

- Figure 1 and thereafter: the definition of “win” is not provided, and readers have to guess what it means.

A definition of a win has been added to the caption.

- The definition of P(DD) is not provided, so readers have difficulty in understanding Figure 2 and Table 3.

A definition of P(DD) has been added to the caption.

- Figure 4 presents a stationary distribution, but it is not clear how the initial condition of the replicator equation was chosen; in fact, because the replicator equation is 203=204-1 dimensional, there should be lots of equilibria in the system and the uniqueness of equilibrium is not at all guaranteed. In that case, the stationary distribution should be highly dependent on the initial condition, but the paper does not consider this problem and only cites “integration technique described in [29]”.

The initial distribution chosen was a uniform distribution of the strategies. This has been clarified in the text.

- It is not clear how many and what sorts of explanatory variables were used for general linear models with recursive feature elimination. Moreover, Tables 4 and 5 seem like just a copy-and-paste from a statistical software, and there are no descriptions about how to read/understand them. Readers have to guess everything, which is apparently a bad sign for a scientific paper. I find this is very critical, as Tables 4 and 5 are one of the main results. Furthermore, there are lots of pieces of information that are not relevant to the discussion in the main text (for example, “Date” and “Time” are obviously not necessary. Most readers could not understand item such as “Omnibus”, “Durbin-Watson”, “Jarque-Bera”, “Cond. No.” and so on, and they are not relevant to or even cited in the main text.)

The date and time have been removed and a few more details have been added to the caption. We purposely included all other summary measures of the linear regression process as good practice in research communication. If the reviewer feels strongly to remove it we will do so but would actually actively encourage all researchers to include as many measures from processes like this as possible to ensure availability and reproducibility of research.

- The fixation probability kappa_1 is discussed in Section 3.3.2 (btw, x_1 in figure panels and in the legend remain uncorrected). However, there is no information about what type of Moran model was employed (e.g. how to transform payoffs to fitness, including the magnitude of selection strength), and only citations to [22] and [24] are given; readers have to look at these in order to figure out what assumptions are actually made. This is again problematic, because Figure 7 is one of the main results, based on which the authors claim that negative skew is a key to evolutionary success. Also, a justification is missing about why taking the average of (N kappa_1) over N=3 to 14 should be an appropriate way to measure evolutionary success.

More explanation has been given here.

- It is not clear why Eq.(23) is the condition for extortion. Eq.(23) means -beta < alpha, which means chi=(-beta)/alpha > 1 for alpha<0, but means chi=(-beta)/alpha < 1 for alpha>0.

We have corrected Eq.(23).

Another issue is that there are lots of grammatical unclarity throughout the manuscript, which prevents readers from smooth reading/understanding. To just list some from the first 3 pages;

- Abstract, “… lack thereof of zero-determinant strategies is done by placing some zero-determinant strategy”: What is the meaning of “the lack of ZD is done by placing ZD”?

We have improved punctuation to hopefully clarify this sentence.

- Abstract: “their extortionateness quantified” -> “their extortionateness is quantified”

The wording has been fixed.

- Section 1, 3rd paragraph: “opponents previous play” -> “opponent’s previous play”
- Section 1, 2nd last paragraph: “in to” -> “into”
- Section 2, 2nd paragraph: “by elements of R^4 mapping” -> “by elements of R^4, mapping”
- Below Eq.(6) “In Section,”: a section number is missing.

These have all been addressed.

- Below Eq.(15) “triangular (15) plane (13) in 3 dimensions (14)”: This is not a reader-friendly way of citing equations.

We have modified this sentence.

- Below Eq.(15) “from [33] which is” -> “from [33], which is”
- Section 2.2, 2nd paragraph: “Table 1 which shows” -> “Table 1, which shows”

These have all been addressed.

Other points I’d like to mention. It is not natural to divide the abstract into two paragraphs. It should be one paragraph. Also, there are too many paragraphs in one section on average. Could you make it lesser overall?

This seemed like a strange request from the original editor. If the reviewer would like us to merge paragraphs we can do so. If not, we would prefer to keep the presentation as is as we feel it improves readability.

## Reviewer 2

1. Missing the reference to the section at the beginning of page 3: “In Section , the reverse problem is considered”

This has been fixed.

2. Equation (15) should be aligned to equations (13) and (14).

The equality signs and strictly less than sign are now all aligned.

3. The second part of equation (24) can be a distinct equation, e.g. (25)

This has been done.

4. Although it is indicated in the title, Figs. 1 and 3 do not have an explicit label for the y-axis. I suggest including SSE as label of the y-axis.

These have been added.

5. A dot is missing in the first sentence of the subsection 3.3.2 before Figure 7. “In [24] a large data set of pairwise fixation probabilities in the Moran process is made available at [22]”

This has been fixed.

6. The quote reported at the end of section 3 could be in italics.

This has been fixed.

7. A comma is missing in the following sentence after “Rather”: “Rather more sophisticated strategies are able to adapt to a variety of opponents and act extortionately only against weaker strategies while cooperating with like-minded strategies that are not susceptible to extortion.”

This has been fixed.
